# Smoking behavior, attitudes, and cessation counseling among healthcare professionals in Armenia

**DOI:** 10.1186/1471-2458-12-1028

**Published:** 2012-11-24

**Authors:** Narine K Movsisyan, Petrosyan Varduhi, Harutyunyan Arusyak, Petrosyan Diana, Muradyan Armen, Stillman A Frances

**Affiliations:** 1 College of Health Sciences, American University of Armenia, Yerevan, Armenia; 2 National Oncology Center, Yerevan, Armenia; 3 Institute for Global Tobacco Control, Bloomberg School of Public Health, Johns Hopkins University, Baltimore, MD, USA

**Keywords:** Smoking cessation, Smoke-free hospital policy, Survey research, Qualitative research, Healthcare professionals, Physician smoking, Armenia, Transition economies

## Abstract

**Background:**

Smoking cessation counseling by health professionals has been effective in increasing cessation rates. However, little is known about smoking cessation training and practices in transition countries with high smoking prevalence such as Armenia. This study identified smoking-related attitudes and behavior of physicians and nurses in a 500-bed hospital in Yerevan, Armenia, the largest cancer hospital in the country, and explored barriers to their effective participation in smoking cessation interventions.

**Methods:**

This study used mixed quantitative and qualitative methods. Trained interviewers conducted a survey with physicians and nurses using a 42-item self-administered questionnaire that assessed their smoking-related attitudes and behavior and smoking cessation counseling training. Four focus group discussions with hospital physicians and nurses explored barriers to effective smoking cessation interventions. The focus group sessions were audio-taped, transcribed, and analyzed.

**Results:**

The survey response rate was 58.5% (93/159) for physicians and 72.2% (122/169) for nurses. Smoking prevalence was almost five times higher in physicians compared to nurses (31.2% vs. 6.6%, p < 0.001). Non-smokers and ex-smokers had more positive attitudes toward the hospital’s smoke-free policy compared to smokers (90.1% and 88.2% vs. 73.0%). About 42.6% of nurses and 26.9% of physicians reported having had formal training on smoking cessation methods. While both groups showed high support for routinely assisting patients to quit smoking, nurses more often than physicians considered health professionals as role models for patients.

**Conclusions:**

This study was the first to explore differences in smoking-related attitudes and behavior among hospital physicians and nurses in Yerevan, Armenia. The study found substantial behavioral and attitudinal differences in these two groups. The study revealed a critical need for integrating cessation counseling training into Armenia’s medical education. As nurses had more positive attitudes toward cessation counseling compared to physicians, and more often reported having cessation training, they are an untapped resource that could be more actively engaged in smoking cessation interventions in healthcare settings.

## Background

According to the World Health Organization (WHO), tobacco is the second major cause of death and the fourth most common risk factor for disease worldwide [1]. According to the WHO Global Status Report on non-communicable disease (NCDs), the European region has the highest overall smoking prevalence rate of 29% [2]. More than 75% of tobacco related deaths will occur in low and middle income countries due to high prevalence of smoking among men [2,3]. The estimated burden of NCDs mortality among persons under the age of 60 was more than twice higher in low than in high income countries (29% vs. 13%) [2]. Interventions by health professionals can be effective in increasing cessation rates [4-7]; however, numerous barriers to implementing tobacco cessation efforts exist in medical settings, including insufficient skills and knowledge and lack of time and incentives [5,6]. Physician smoking may be another barrier for effective cessation interventions [8]. As a role model, physicians, if they are not smokers, could best persuade patients to quit [9].

Promisingly, a number of studies showed that smoking prevalence among medical doctors in high income countries such as the US, the UK, or Scandinavian countries have fallen sharply during the last decades of the 20^th^ century [9-11]. Little is known, however, about smoking behavior, attitudes, and smoking cessation training and practices of healthcare practitioners in low and middle income countries. Data from the Global Health Professions Student Survey 2005–2007 (GHPSS) showed that the use of tobacco remained widespread (up to 40%) among medical, dental, pharmacology and nursing students in many Eastern European countries and formal training on smoking cessation was lacking in the majority (25/31) of surveyed countries [12].

Armenia, an economy in transition located in Eastern Europe, has high smoking prevalence that contributes to thousands of preventable deaths from NCDs each year. Among men, the smoking rate is one of the highest in the European region while women smoke far less than men (2.1% vs. 59.6%) [13]. Similar male–female smoking prevalence differences were reported for other transition countries, and are typical for many low income countries [2]. According to Armenia’s national legislation, indoor smoking is banned in health, education, culture facilities; however, compliance with the existing legislation has been inadequate due to lack of enforcement mechanisms, lack of awareness about the policy among the administrators of those facilities and the general public, and lack of awareness about the harms of smoking and secondhand smoke, and societal tolerance towards smoking [14,15] . Furthermore, smoking among Armenian physicians is remarkable. Perrin et al. found that 12.8% of female and 48.5% of male physicians were current smokers in Yerevan, Armenia [16]. This finding is consistent with the findings from the GHPSS on smoking among medical students, according to which 7.7% of female and half of male medical students smoked [12].

In this study we aimed to: (1) explore smoking-related attitudes and behavior of physicians and nurses in the largest cancer care center in Armenia, and (2) provide insights into existing barriers to effective participation of health professionals in smoking cessation interventions.

## Methods

### Setting

The study was conducted in a 500-bed tertiary referral hospital located in the capital city Yerevan that provides comprehensive cancer care that is publicly financed. This formative research was implemented within the framework of a larger demonstration project to implement smoke-free interventions in hospitals and universities in Yerevan, Armenia and assess the effectiveness of those interventions.

### Ethical approval

The Institutional Review Boards of the American University of Armenia and Johns Hopkins University Bloomberg School of Public Health reviewed and approved the study protocol.

### Study population

The study population included full or part-time physicians (including medical residents), and nurses who were available at the time of the survey. Non-clinical staff, such as medical equipment maintenance and administrative personnel, and the ancillary staff were not included in this analysis.

The study used quantitative (survey) and qualitative (focus groups discussions) methods to address the research questions.

### Survey

#### Survey instrument

We used a self-administered questionnaire developed by the Institute for Global Tobacco Control team at Johns Hopkins University [17]. The 42-item survey questionnaire included (but was not limited to) standardized questions on socio-demographic variables and smoking status, smoking behavior (e.g., number of cigarettes smoked at work), attitudes toward smoke-free policy and its implementation (Likert scale), attitudes toward clinician’s role in smoking cessation, and whether they received a relevant training (“Yes/No” answers).

Self-reported smoking status was measured as a categorical variable, with “current smoker” defined as having smoked 100 cigarettes in lifetime and currently smoking (daily or occasional). “Ex-smoker” was defined as having smoked 100 cigarettes in lifetime and not smoking presently and “never smoker” as having smoked less than 100 cigarettes in lifetime.

#### Survey data collection and analysis

Trained interviewers conducted the survey in June-July 2009. The interviewers contacted the available clinical staff (additional visits were made to cover all the shifts), explained the study aims and procedures, and asked for their consent to participate. The participants returned self-administered questionnaires in a sealed envelope. The collected data were entered, cleaned, and analyzed using SPSS and STATA statistical packages.

The study generated descriptive statistics. Differences between nurses and physicians were compared using chi-square statistic for categorical variables and Student’s t-test for continuous variables.

#### Survey participants

The study team attempted to contact all physicians and nurses working in this tertiary hospital (census). Ninety three physicians and 122 nurses returned the completed questionnaires. The survey response rate was 58.5% (93/159) for physicians and 72.2% (122/169) for nurses. Thus, this study had more than 90% power for detecting a true difference in smoking prevalence between the two occupational groups (physicians and nurses).

The age distribution for responding physicians and nurses were similar, the mean age was 42.3 ± 11.0 for physicians and 40.3 ± 12.3 for nurses (Table 1). While the majority of nurses were females (98.4%), an equal proportion of male and female physicians participated in the survey. About one-third of physicians (29.0%) had or were studying toward PhDs.

**Table 1:** The survey respondents’ age and gender by occupation

	**Nurses % (N)**	**Physicians % (N)**	**p-value**
	**(n = 122)**	**(n = 93)**	
Gender			<**0**.**001**
Male	1.6 (2)	50.5 (47)	
Female	98.4 (120)	49.5 (46)	
Age (yrs), mean ± sd	40.3 ± 11.0	42.3 ± 12.3	0.243

### Focus group discussions

Following the completion of the survey, the study team conducted four focus group discussions (FGDs) with hospital physicians and nurses for in-depth exploration of the existing and potential barriers to the provision of smoking cessation assistance to patients in the hospital.

#### FGDs recruitment

The study team recruited FGDs participants using a snowball approach where contacts established during the survey served as a starting point for recruiting additional participants. The balance across various age groups and hospital departments was carefully maintained to ensure diversity in the groups.

#### FGDs data collection and analysis

A semi-structured guide was developed to assist in the discussion process. The guide included open-ended questions on rights of non-smokers and smokers, attitudes towards smoke-free policies and enforcement of the smoke-free policy in the hospital, the role of health professionals in assisting patients to quit, knowledge of contemporary approaches in smoking cessation, and readiness to help patients in quitting smoking. Trained facilitators moderated the FGDs, which were audio taped with the consent from the participants. All participants completed a brief questionnaire on age, gender, occupation and smoking status. The study team transcribed the discussions verbatim and the transcripts were reviewed and coded according to the initial themes covered by the guiding questions. After the initial coding was completed, the qualitative analysis focused on the following themes: beliefs about smoking addiction and attitudes toward worksite smoking, the role of health professionals in assisting their patients to quit, the awareness and use of smoking cessation methods. The second review identified new themes: use of electronic cigarettes and issues in clinical management of cancer patients.

#### FGDs participants

The study team conducted focus group sessions with 10 physicians and 13 nurses at the hospital: one with nurses, two with doctors, and one with mixed composition. Among eighteen female participants, three were smokers (two doctors, one nurse). Among five male physicians, two were smokers, two were ex-smokers, and one was a non-smoker.

## Results

### Survey results

#### Smoking status and behavior

Smoking prevalence among health professionals was 17.2% (95% CI: 12.1-22.3). Current smoking was significantly more prevalent in male physicians compare to their female counterparts (42.6% vs. 19.6%, p < 0.001). Smoking was approximately 5 times more prevalent among physicians than among nurses (31.2% vs. 6.6%). The duration of smoking (years) and the number of cigarettes smoked per day was similar among smokers in both professions (Table 2). However, nurses smoked significantly less during work hours (p = 0.05).

**Table 2:** The survey respondents’ smoking status and behavior by gender and occupation

	**Nurses % (N)**		**Physicians % (N)**		**p-value**
	**(n = 122)**		**(n = 93)**		
	**Male**	**Female**	**Male**	**Female**	
Smoking status					<**0**.**001**
Daily	-	4.2 (5)	34.0 (10)	10.9 (5)	
Occasional	-	2.5 (3)	8.5 (16)	8.7 (4)	
Ex-smoker	-	1.7 (2)	21.2 (4)	10.9 (5)	
Never smoked	100 (2)	91.7 (100)	36.2 (17)	69.6 (32)	
Smoking duration (yrs), mean ± sd	15.6 ± 8.2		16.8 ± 10.7		0.743
Cigarettes/day, mean ± sd	6.7 ± 7.2		17.5 ± 15.2		0.106
Cigarettes/day at work, mean ± sd	1.0 ± 2.6		7.4 ± 8.0		**0**.**046**
Quit attempts in 30 days, %(N)	16.7 (1)		34.8 (8)		0.393

#### Attitudes toward smoke-free hospital policies

The survey participants showed strong support toward smoke-free hospital policies (Table 3), with the highest percent of agreement on the item “A hospital should be a smoke free environment” and the least support for the item “A smoking ban would be unfair to smokers.” However, 62.3% of nurses and 64.5% of physicians thought that such policies would be difficult to implement (Table 4). Nurse and physician participants responded similarly to all attitudinal statements, except the potential impact of smoke-free policy on the quality of care: significantly higher proportion of nurses believed that smoke-free policies could improve patient care (68.9% vs. 55.9%, p = 0.05).

**Table 3:** The attitudes (% agreement) toward the smoke-free hospital policy by occupation

**Statement**	**Nurses % (N)**	**Physicians % (N)**	**p-value**
1. A hospital should be a smoke free environment.	91.8 (112)	90.3 (84)	0.705
2. Tobacco smoke is dangerous for nonsmokers’ health.	91.0 (111)	91.4 (85)	0.916
3. I would like/am happy to see this hospital become smoke-free.	87.7 (107)	86.0 (80)	0.716
4. A smoking ban would be unfair to smokers.	29.5 (36)	30.1 (28)	0.924
5. Smoking is dangerous to smokers’ health.	91.8 (112)	90.3 (84)	0.705
6. A smoke-free hospital would improve the quality of care the patient receives.	68.9 (84)	55.9 (52)	**0**.**051**
7. The smoking habits of employees of this hospital influence others.	35.3 (43)	45.2 (42)	0.141
8. A smoke-free policy is difficult to enforce.	62.3 (76)	64.5 (60)	0.738

**Table 4:** The attitudes (% agreement) toward the smoke-free hospital policy by smoking status

**Statement**	**Non**-**smokers% (N)**	**Current smokers% (N)**	**Ex-smokers% (N)**	**p-value**
1. A hospital should be a smoke free environment.	91.9 (148)	86.5 (32)	94.1 (16)	0.521
2. Tobacco smoke is dangerous for nonsmokers’ health.	91.6 (141)	85.3 (29)	93.8 (15)	0.475
3. I would like/am happy to see this hospital become smoke-free.	90.1 (145)	73.0 (27)	88.2 (15)	**0**.**020**
4. A smoking ban would be unfair to smokers.	28.0 (45)	40.5 (15)	23.5 (4)	0.269
5. Smoking is dangerous to smokers’ health.	93.2 (150)	81.1 (30)	94.1 (16)	**0**.**059**
6. A smoke-free hospital would improve the quality of care the patient receives.	65.2 (105)	51.4 (19)	70.6 (12)	0.233
7. The smoking habits of employees of this hospital influence others.	41.0 (66)	35.1 (13)	35.3 (6)	0.752
8. A smoke-free policy is difficult to enforce.	62.1 (100)	64.9 (24)	70.6 (12)	0.768

The attitudes toward smoke-free policies among current, never and former smokers differed on two of the eight statements. Thus, only about 73.0% of the current smokers liked the idea of implementing smoke-free policy in the hospital; significantly less than non-smokers (90.1%) and ex-smokers (88.2%). Current smokers less often agreed that smoking was dangerous to smokers’ health (81.1%) compared to non-smokers (93.2%) and ex-smokers (94.1%).

#### Attitudes toward health professional’s role in smoking cessation

The attitudes of physicians and nurses on smoking cessation issues were slightly different from each other (Table 5). In particular, nurses agreed more often that trainings on cessation methods were necessary and that health professionals were role models for patients and the public; however, both groups agreed on the necessity to routinely advise patients to quit.

**Table 5:** The attitudes toward cessation advice and reported training by occupation

**Question**	**% (N) who answered “Yes”**		**p-value**
	**Nurses**	**Physicians**	
Should health professionals get specific training on cessation techniques?	58.2 (71)	45.2 (42)	**0.058**
Do health professionals serve as “role models” for their patients and the public?	74.6 (91)	62.4 (58)	**0.054**
Should health professionals routinely advise their patients who smoke to quit smoking?	90.2 (110)	87.1 (81)	0.479
During your (medical, dental, nursing or pharmacy) school training, were you taught in any of your classes about the dangers of smoking?	66.4 (81)	62.4 (58)	0.540
During your (medical, dental, nursing or pharmacy) school training, have you ever received any formal training in smoking cessation approaches to use with patients?	42.6 (52)	26.9 (25)	**0.017**

The attitudes toward health professional’s role in smoking cessation differed by the respondent’s smoking status: non-smokers and ex-smokers agreed more often than current smokers that health professionals should get training on smoking cessation methods (57.1% and 47.1% vs. 35.1%, p < 0.05) and should routinely advise smoking patients to quit (92.6% and 82.4% vs. 75.7%, p < 0.01).

#### Smoking cessation training

About 42.6% of nurses and 26.9% of physicians reported having a formal training on smoking cessation methods (p = 0.02).

### Focus group discussions results

#### Beliefs about smoking addiction and attitudes towards worksite smoking

Smoking was often seen as a habit not an addiction. Many suggested that if a person wanted to quit smoking, s/he could do it without any additional assistance. Few participants acknowledged that smokers might not be able to quit on their own because of dependence. Though common, worksite smoking reportedly was limited to private rooms. A sizable number of nurses believed that “the majority of doctors are smokers”. This was seen as a barrier to implementation of smoke-free policy in the hospital since “the subordination and respect to physicians” prevents them from asking physicians not to smoke at work.

#### Beliefs about the role of health professionals in patient’s quitting

When asked about the role of health professionals in patients’ smoking cessation, nurses underscored that doctors are more influential in changing patients’ behavior. However, they admitted that the nurse’s role also was important, especially in patient education. While the nurses viewed doctors as a role model, only a few oncologists supported this idea. The majority of doctors claimed having no role in a patient’s quitting.

"“A person should make a conscious decision to quit smoking. It should be only his/her decision. I don’t really think that a medical worker’s role is very important in this issue.” [MD/female/non-smoker]"

"“They (doctors) have no role. Very few physicians are advising their patients to quit smoking and many physicians even allow their patients to smoke.” [MD/male/ex-smoker]"

The physicians suggested several explanations to support their position. Some pointed out that quitting smoking did not help a cancer patient but put him/her under additional stress. Others felt uncomfortable intervening as they were smokers themselves. Several reiterated that quitting smoking did not require any additional assistance or intervention.

"“It is true that cancer and smoking are strongly interconnected but if a person is already ill with 4^th^ stage cancer then prohibiting smoking only causes more stress for the person. It sounds strange but oncologists have no role in patient’s smoking cessation because even if they see a patient in the 1^st^ stage of illness then the process has already begun and prognosis for a disease is not being changed because of quitting. For instance, cardiologists could have a huge role in patients’ smoking cessation because the prognosis for cardiovascular diseases changes after quitting.” [MD/male/ex-smoker]"

#### Awareness and use of smoking cessation methods

Awareness of smoking cessation pharmacological aids and counseling methods was limited. When prompted, several mentioned nicotine replacement products, such as gums and patches, and other pharmacological products, such as Tabex (Cytisine) and Zyban (Bupropion). A physician, who was a smoker herself, noted a crucial lack of information about smoking cessation aids. A few physicians mentioned trying several of the aids and felt that they were ineffective. Several nurses reported that their husbands had tried nicotine gums or patches. Besides the listed cessation tools, participants mentioned the use of electronic cigarettes as a device perceived as a less harmful alternative to the conventional cigarette. Allen Carr’s book [18] was very popular among ex-smokers who succeeded to quit with help of this book as well as among those who failed. None of the participants mentioned counseling as a smoking cessation assistance tool.

#### Issues in clinical management related to smoking

An unexpected revelation regarding perceived evidence in the clinical management of patients who were current smokers emerged in the focus group discussions. Many of the doctors and nurses stated that allowing “just one cigarette” would prevent lung congestion in post-operative cancer patients with a long history of smoking and could be beneficial for the patients. Some also feared that abrupt quitting could be dangerous for some patients and not helpful for others.

"“We explain to patients, that it is forbidden to smoke after the surgery. However, after the surgery we give a patient one cigarette to make him/her to cough in order to clean lungs from sputum. Smoke stimulates the coughing reflex.” [Nurse/female/non-smoker]"

"“When you quit smoking you start to have coughing. A person with central lung cancer can die from severe blood loss because of this cough.” [MD/male/smoker]"

## Discussion

This study’s findings are based on data from health professionals working in one large hospital, and therefore, may not be representative of all healthcare providers in Armenia. The anonymous questionnaire limited a social desirability bias, however, the extent of over-reporting of positive attitudes and under-reporting of smoking behavior cannot be assessed. The research team pre-tested a survey instrument adapted from one used elsewhere, but its validity in the study population has not been studied formally. The participation in the survey was high and the response rates are comparable with other studies in this population [11].

Furthermore, the study was conducted in the largest cancer hospital in the country, where one would presume that the providers have the most knowledge of and first-hand experience with the consequences of smoking. The combination of quantitative and qualitative methods enriched our understanding of the survey results and helped to reveal unforeseen issues related to postoperative care of cancer patients.

Similar to the results of Perrin et al. [16], worksite smoking was normative among the doctors; however, it was limited to physicians’ offices. Nurses smoked fewer cigarettes at work than physicians, likely due to the lack of private space and time afforded. The survey showed that nurses more often reported to receive smoking cessation training and they were more likely to consider health professionals as role models for patients and the public than physicians. Nurses and physicians of the oncology center strongly supported and shared the view that health professionals in general should routinely advise their patients to quit smoking. However, the qualitative study demonstrated that physicians were likely to underestimate the value of their own advice and participation in smoking cessation counseling and they were more prone to delegating the counseling role to other specialists, such as primary health care providers or cardiologists. This lack of oncologists’ interest to be closely involved in smoking cessation assistance could be explained by lack of appropriate training on smoking cessation counseling along with lack of time and incentives as suggested by both qualitative and quantitative findings.

More nurses than physicians reported having received training on smoking cessation. This is consonant with the earlier study by Warren et al. that found a similar gap in the reported smoking cessation training among nursing (43.1%) and medical (32.3%) students in Armenia [12]. Furthermore, more than a third of physicians in our study reported not being taught about risks of smoking in a medical school which calls for a critical review of the current medical training curriculum in Armenia.

While skeptical of its effectiveness, both the survey and FGDs demonstrated that both nurses and physicians supported indoor smoking ban in the hospital. Current smokers, understandably, were not as supportive of the smoke-free policy and were less willing to assist smokers in quitting; these findings were consistent with other studies that reported about association between the smoking status and attitudes towards smoke-free policies [19-22]. The findings of this study also indicate about a very early stage in the movement for smoke-free hospitals in Armenia that could be compared, for example, to the situation in the US in late 80s prior to adopting standards for smoke-free hospitals by the Joint Commission on Accreditation of Healthcare Organizations [23-25]. Presently, hospitals in high income countries are making a transition from indoor smoking ban toward smoke-free campuses [26,27]. Such a transition is not feasible yet in Armenia where implementing hospital indoor smoking ban has been a challenge.

Another important, unexpected, finding was the knowledge gap related to tobacco addiction and treatment of nicotine dependence among these health professionals. The oncologists questioned the rationale for smoking cessation efforts targeting cancer patients. Advising a patient in intensive care to light a cigarette was a common practice and believed to be a measure to prevent post-surgical complications. This finding confirms previous assertions that the quality of cancer care in Armenia needs more attention and improvement [28].

Smoking remains normative not only in population at large but also among Armenian physicians [13,16]. While lower than the population prevalence in general, physicians’ smoking prevalence was almost five times that of the nurses. Part of this disparity reflects the wide gender differences in smoking prevalence rates among health care providers (44.6% for male physicians, 19.7% for female physicians, 6.5% for female nurses) as well as the general population (59.6% males, 2.1% females) [11]. These results are consistent with Perrin et al. (2004) findings on physicians’ smoking in Yerevan, Armenia [16]. However, the patterns of smoking among physicians and nurses in our study are different from the trends observed in many high income countries, where the smoking prevalence is higher among nurses compared to physicians [15,18,21,28,29]. The low smoking prevalence of 6.5% among Armenian nurses in this study is still 3 times higher than the general rate for women and also over two times higher than the 2006 Global Survey of Nursing Students that reported a smoking prevalence of 2.4% for female nurses in Armenia, the lowest of the ten countries surveyed in the European region [30]. However, smoking prevalence in male physicians was much lower than in the general male population, suggesting that socio-cultural influences outside the scope of this study could be driving the relationships between smoking behavior, gender, and occupation.

This study adds to our knowledge of the barriers to more effective participation of health professionals as key players in smoking cessation efforts in Armenia and other similar economies in transition. Healthcare professionals in high income countries were the first to quit, paving the way for others as role models and as advocates for environmental and policy change [8,9,11]. No such trend is yet apparent in Armenia. Our study identified a critical lack of appropriate knowledge and skills needed to help patients quit. It also revealed that oncologists’ motivation to personally serve as a role model for patients was low; this finding needs to be considered in a broader context such as normalcy of smoking behavior in the society and overload and stressful work environment for cancer care providers. All mentioned above are only a few of the challenges to building capacities for smoking cessation services within the Armenian healthcare system.

## Conclusions

The study was the first to explore the differences in smoking-related attitudes and behavior among the hospital physicians and nurses in Armenia and found substantial behavior and attitudinal differences in these two groups.

The findings indicate a critical need for raising healthcare providers’ preparedness for implementing smoking cessation interventions in hospital settings in Armenia and other economies in transition facing similar issues. Based on the evidence that nurses had more positive attitudes on cessation counseling compared to physicians and more often reported having a training on cessation approaches, we conclude that nurses have been an untapped resource to be more actively engaged in smoking cessation interventions in healthcare settings.

## Competing interests

Authors declare no competing interests.

## Authors’ contributions

NM conceptualized the scope of this paper, performed the analysis and drafted the manuscript. AH and DP coordinated the data collection and participated in the data analysis, VP critically revised the draft manuscript and made substantial contribution to its finalization. AM and FS reviewed and contributed to the manuscript. All authors approved the final manuscript.

## Pre-publication history

The pre-publication history for this paper can be accessed here:

http://www.biomedcentral.com/1471-2458/12/1028/prepub
